# Current Approaches to and Future Perspectives on Methomyl Degradation in Contaminated Soil/Water Environments

**DOI:** 10.3390/molecules25030738

**Published:** 2020-02-08

**Authors:** Ziqiu Lin, Wenping Zhang, Shimei Pang, Yaohua Huang, Sandhya Mishra, Pankaj Bhatt, Shaohua Chen

**Affiliations:** 1 State Key Laboratory for Conservation and Utilization of Subtropical Agro-bioresources, Guangdong Province Key Laboratory of Microbial Signals and Disease Control, Integrative Microbiology Research Centre, South China Agricultural University, Guangzhou 510642, China; 20192047010@stu.scau.edu.cn (Z.L.); 20191047008@stu.scau.edu.cn (W.Z.); 20192047012@stu.scau.edu.cn (S.P.); 20183138021@stu.scau.edu.cn (Y.H.); sandhyamanshi@gmail.com (S.M.); pankajbhatt.bhatt472@gmail.com (P.B.); 2 Guangdong Laboratory of Lingnan Modern Agriculture, Guangzhou 510642, China

**Keywords:** methomyl, biodegradation, physicochemical degradation, mechanism, degradation pathway

## Abstract

Methomyl is a broad-spectrum oxime carbamate commonly used to control arthropods, nematodes, flies, and crop pests. However, extensive use of this pesticide in agricultural practices has led to environmental toxicity and human health issues. Oxidation, incineration, adsorption, and microbial degradation methods have been developed to remove insecticidal residues from soil/water environments. Compared with physicochemical methods, biodegradation is considered to be a cost-effective and ecofriendly approach to the removal of pesticide residues. Therefore, micro-organisms have become a key component of the degradation and detoxification of methomyl through catabolic pathways and genetic determinants. Several species of methomyl-degrading bacteria have been isolated and characterized, including *Paracoccus*, *Pseudomonas*, *Aminobacter*, *Flavobacterium*, *Alcaligenes*, *Bacillus*, *Serratia*, *Novosphingobium*, and *Trametes*. The degradation pathways of methomyl and the fate of several metabolites have been investigated. Further in-depth studies based on molecular biology and genetics are needed to elaborate their role in the evolution of novel catabolic pathways and the microbial degradation of methomyl. In this review, we highlight the mechanism of microbial degradation of methomyl along with metabolic pathways and genes/enzymes of different genera.

## 1. Introduction

**Figure 1:**
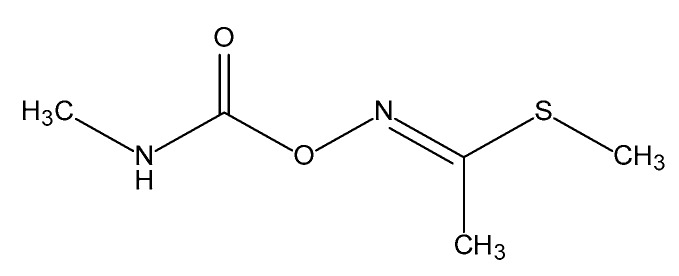
The chemical structure of methomyl.

Carbamate insecticides are commonly used in various agricultural sectors, particularly crop protection. The higher global demand for pesticides has created a market that is worth billions of dollars. Carbamates have emerged as a better substitute for organophosphorus pesticides due to their broad-spectrum efficacy and short residual period [1,2]. Methomyl (S-methyl-N-(methylcarbamoyloxy)-thioacetimide) (MET) (Figure 1), an oxime pesticide in the carbamate class, is widely used to control the eggs, larvae, and adults of different pests [3]. Methomyl inhibits acetylcholinesterase activity, causing a nerve tissue failure that kills insects [4,5,6]. However, long-term applications of methomyl have resulted in the development of resistance in some insects [7,8,9].

**Figure 2:**
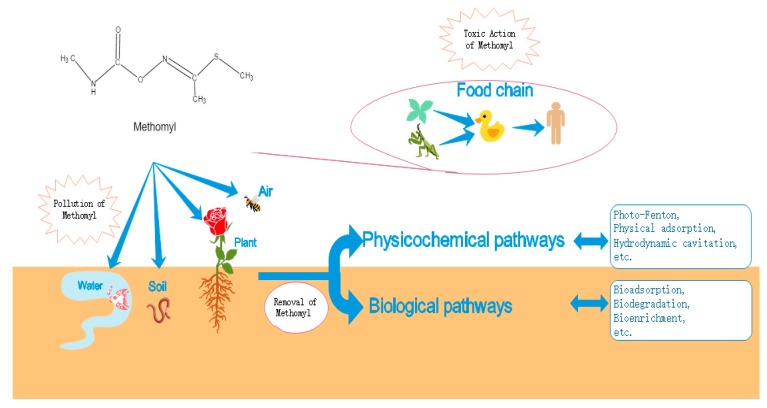
Contamination and removal of methomyl from soil environments.

Approximately 10% of the applied pesticide reaches target organisms, and the remaining 90% is distributed in the environment where it can adversely affect non-target organisms and ecosystems [10]. Due to its high water solubility (57.9 g L^−1^, 25 °C), methomyl in the environment cannot be fixed in the soil [11]. The half-life of methomyl ranges between 3 and 50 days in soil, between 6 and 262 days in water, and between 160 and 224 days in air [12,13,14]. Environmental residues of methomyl can affect non-target organisms through the air, water, soil, and food chain (Figure 2). Long-term exposure to methomyl can result in hepatotoxicity, cytotoxicity, and neurotoxicity in animals [15,16,17]. According to a survey conducted in France, 4.2% of the population was directly or indirectly poisoned with methomyl during the period 2012–2016 [18]. It has been detected in the blood, liver, kidneys, and brain of humans and animals [19,20,21]. Methomyl has been banned in many European countries due to its extremely high residual toxicity towards mammals, birds, and the environment [18]. Spraying, leaching, sorption, and volatilization can result in the contamination of ecosystems. Therefore, there is an urgent need to remove residual methomyl from the environment.

Different degradation processes for the decontamination of methomyl-affected environments have been tested. Physicochemical methods, such as adsorption, oxidation, the Photo-Fenton process, ultrasound cavitation (US), and hydrodynamic cavitation (HC), have been studied extensively [22,23,24]. Microbial degradation of methomyl has emerged as a potential tool for the large-scale removal of this contaminant from the environment. A few reports focus on the isolation and characterization of methomyl-degrading micro-organisms. These microbes include *Paracoccus*, *Pseudomonas*, *Aminobacter*, *Flavobacterium*, *Alcaligenes*, *Bacillus*, *Serratia*, *Novosphingobium*, and *Trametes* [25,26,27,28,29]. Microbial degradation was found to be ecofriendly and acceptable for large-scale bioremediation of methomyl-contaminated sites [30,31,32,33]. In addition, the degradation pathways of methomyl and the fate of several metabolites have been investigated. However, there is a limited number of studies on methomyl-degrading enzymes and the corresponding genes in microbes. Furthermore, few reviews focus on the mechanisms and degradation pathways of methomyl. Therefore, the purpose of this review is to summarize methomyl degradation mechanisms and analyze the bioremediation potential of methomyl-degrading microbes in contaminated soil/water environments.

## 2. Toxicological Effects of Methomyl Insecticides

The chemical structure of methomyl is unstable, and it is easily decomposed in the environment. However, the use of methomyl has exceeded its natural degradation rate, leading to a cumulative effect on ecosystems and organisms [34]. The toxicological impacts of methomyl on aquatic animals, amphibians, land mammals, and humans are presented in Table 1.

Different aspects of methomyl toxicity on tilapia as a model aquatic organism have been studied [15,35,36]. A high concentration of methomyl was found to drastically change biochemical and histological activities in tilapia. Islamy et al. [35] reported that genotoxicity increased as the concentration of methomyl increased (0-10 mg L^−1^). Moreover, prolonged exposure to methomyl at a concentration above 20 mg L^−1^ can result in injury to testicular tissue [36,37]. Several studies have demonstrated that higher concentrations of methomyl might be responsible for the disruption of the endocrine system and expression of the *LHR*, *StAR*, *3β-HSD*, and *ARα* genes in testes and the *LHβ* gene in the pituitary. Meng et al. [38] reported significantly reduced expressions of these genes at higher methomyl concentrations. Hazardous effects of methomyl have also been studied in frogs and toads as virulence testers and representatives of amphibians [39,45,46]. Short-term exposure to methomyl can severely affect the survival rate of tadpoles by causing deformations, intestinal contortions, a loss of appetite, and hyper-activation. Prolonged exposure to methomyl can cause a contortion of the spinal cord and a reduction in muscle carbohydrates [47]. Sub-lethal concentrations of methomyl can result in cell damage, an increased stress response in the liver, and repressed growth in frogs [16].

Methomyl significantly inhibits acetylcholinesterase activity in mammals and causes various health hazards related to neural, muscular, genital, intestinal, and reproductive functions. Mahgoub and El-Medany [40] reported that long-term exposure to methomyl can lead to testicular and liver damage in rats and inhibits the activity of the brain, erythrocytes (RBC), and cholinesterase (ChE). The LC_50_ value of methomyl for experimental rats is 20 mg L^−1^; however, daily feeding of male rats with 1.0 or 0.5 mg (kg·bw)^−1^ of methomyl produces serious reproductive toxicity. It decreases the quality of testicles, seminal vesicles, and the prostate and sperm concentration, sexual potency, and serum testosterone levels [41,42].

Methomyl is highly toxic to the human body and direct or accidental exposure to high concentrations can result in severe poisoning or death [18,48]. Methomyl has been detected in the stomach, peripheral blood, brain, and heart of factory workers and farmers who are frequently exposed to high concentrations of methomyl. Higher concentrations of methomyl can cause death [20]. An agricultural worker reportedly died after inhaling a heavy dose of methomyl while flying a pesticide-spraying aircraft [19]. A higher concentration of methomyl has also been reported to cause cortical blindness [21]. Moreover, a large number of studies have shown that methomyl can induce DNA damage and apoptosis in HeLa cells and HEK293 cells [44].

## 3. Physicochemical Methods for the Remediation of Methomyl-Affected Environments

**Figure 3:**
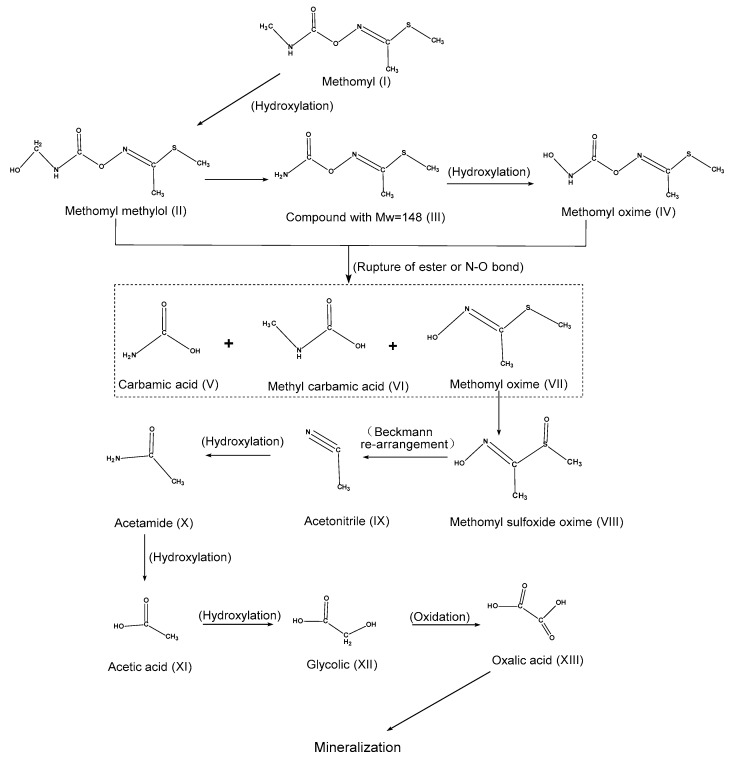
Methomyl degradation pathways by physicochemical methods, adapted from [24,65].

Physicochemical methods have been developed for the large-scale removal of methomyl from contaminated environments (Table 2). In general, these methods are effective but they are expensive to use. Overuse of methomyl can contaminate environmental matrices and exert a variety of toxic effects on humans and aquatic and terrestrial organisms. Thus, it is very important to remove residual methomyl from contaminated environments [47]. Physical adsorption and chemical degradation are the primary techniques for the degradation of pesticides. Other conventional methods for the decontamination of pesticide-polluted sites include activated carbon, UV, TiO_2_, H_2_O_2_, and O_3_ adsorption [49,50,51]. Advanced oxidation processes (AOPs), which are formed by the combination of several oxidants, have been successfully applied to remove various pollutants from the environment [52,53,54,55,56]. AOPs, including the Photo-Fenton, UV/TiO_2_, H_2_O_2_/HC, and Fenton/H_2_O_2_ processes, are considered to be the most efficient chemical degradation methods that consist of multiple oxidants (Figure 3) [22,23,24,57]. Activated carbon is an excellent substituent for the adsorption of methomyl. Cotton-stalk-activated carbon (CSAC) can adsorb 72.85 mg g^−1^ of methomyl at 25 °C [58]. The addition of O_2_, O_3_, and H_2_O_2_ to a methomyl solution can generate hydroxyl radicals that possess a reduction capacity of 2.80 V and can efficiently oxidize pollutants [57]. A DSA Ti/RuO_2_ electrode can degrade approximately 90% methomyl in half an hour under optimal environmental conditions [59]. Methomyl can also directly absorb UV light; however, Tamimi et al. [60] noted that UV irradiation only degraded 4% methomyl in 45 min because of the lower methomyl extinction coefficient at wavelengths higher than 290 nm [60]. However, the combination of UV light with other oxidants, such as in the H_2_O_2_/UV, Fenton/UV, and O_3_/UV systems, significantly enhanced the degradation rate. In these systems, UV absorption by methomyl promotes the formation of super-strong hydroxyl radicals, in the form of H_2_O_2_, Fe(OH)^2+^, and O_3_, respectively, that play an important role in the oxidation of pollutants [22,61]. Sunlight or visible light can also promote the production of hydroxyl radicals by a Fenton reaction for the photocatalytic degradation of methomyl. The light-sensitive point of a Fenton reagent is as high as 600 nm [62]. The UV/TiO_2_ system is the best UV–oxidant system as the absorption value of TiO_2_ is greater than 390 nm and the anatase has a band gap energy of 3.2 eV [60,62]. An addition of CdSO_4_ nanoparticles to a UV/TiO_2_ system can make it more powerful [63].

Hydroxyl radicals can effectively degrade methomyl, which is unstable and highly reactive [52]. In the Fenton/H_2_O_2_ system, both Fe(OH)^2+^ and H_2_O_2_ can produce a large number of hydroxyl radicals by the cleavage of the molecules [43]. The addition of UV to this system results in photo-decarboxylation by Fe(OH)^2+^ ions that promotes the formation of hydroxyl radicals [43]. In the Fenton/Fe-ZSM-5 zeolite system, 16.22 mg L^−1^ of methomyl was completely photodegraded by 5 g L^−1^ of Fe-ZSM-5 zeolite [57]. In the Fenton/humic acid (HA) system, HA promotes the catalytic generation of hydroxyl radicals by reducing Fe^3+^ to Fe^2+^ to improve the system’s degradation efficiency [64].

Ultrasound (US) and hydrodynamic cavitation (HC) are new oxidation technologies that not only produce a variety of oxidizing ions, but also provide a thermal and turbulent environment with a higher efficiency than other Fenton systems [67,68,69]. Application of the Photo-Fenton/US system for the removal of pesticides at large scales is highly beneficial as it can reduce the cost by approximately 98 times when compared to conventional technologies [24].

These technologies can be successfully applied to the treatment of methomyl-contaminated sites. However, it is necessary to develop a treatment technology that is more feasible, ecofriendly, and easy for farmers to apply, requires less chemicals and space, and ensures that pesticides degrade completely [70]. Therefore, a more suitable and advanced degradation technology should be taken into account to increase the ecological and economical safety of the environment.

Methomyl degradation products and pathways have been explored [24,57,59]. The degradation of methomyl occurs relatively slowly under natural conditions. However, it can be completely mineralized into a harmless inorganic substance under catalytic conditions. The most important methomyl degradation pathways are hydroxylation, oxidation, and the cleavage of ester bonds, C-N bonds, and N-O bonds. Initially, methomyl (Ⅰ) hydroxylates to methomyl methylol (Ⅱ), which is subsequently decarboxylated to intermediate products (Ⅲ). Then, a hydroxyl group replaces the H atom of the product (Ⅲ) to form methomyl oxime (Ⅳ). Meanwhile, the cleavage of an ester bond or an N-O bond of organic matter (Ⅰ, Ⅱ, Ⅲ, Ⅳ) produces intermediate products such as carbamic acid (Ⅴ), methyl carbamic acid (Ⅵ), and methomyl oxime (Ⅶ). Product (Ⅶ) soon converts into acetonitrile (Ⅷ) by Beckman rearrangement. Acetonitrile (Ⅷ) finally produces CO_2_, H_2_O, and NO_3_^−^ after a series of oxidation reactions and a hydroxylation translation. In addition, SO_4_^2^^−^ is also produced [66].

Reactions involved in the degradation of methomyl molecules in the atmosphere, possible degradation processes, and the influence of temperature on degradation have been studied by establishing a potential energy surface [14]. Degradation of methomyl in the atmosphere was found to include an H atom extraction reaction and a hydroxyl radical addition reaction. These reactions took place in different groups and produced various intermediate products; however, the study could not determine the final inorganic products. Extraction reactions and addition reactions are easily affected by temperature; a rise in temperature promotes addition reactions and reduces the effectiveness of extraction reactions [14]. The addition of an Fe-zsm-5 zeolite catalyst during the breaking of an ester bond or an N-O bond of organic matter can also generate CO_2_ and H_2_O (Ⅰ, Ⅱ, Ⅲ, Ⅳ). It was inferred that N atoms form NH_4_^+^ and NO_2_^−^ when removed from methomyl [57]. By comparing changes in NO_3_^−^, NH_4_^+^, and NO_2_^−^ during the degradation process, another study proved that NH_4_^+^ and NO_2_^−^ finally generate NO_3_^−^ [66].

## 4. Microbial Degradation of Methomyl

Microbial degradation is a potential approach to the decontamination of pesticide-polluted sites. Compared with physicochemical methods, microbial degradation is considered to be a cost-effective and ecofriendly approach to the removal of pesticide residues [31,32,33]. Biodegrading micro-organisms, including bacteria, fungi, actinomycetes, and algae, can be obtained by enrichment cultures, genetic modification, or gene cloning [70,71,72,73]. Researchers have developed an enrichment culture technique to isolate methomyl-degrading micro-organisms from sewage treatment systems, irrigation areas, and volunteers’ stool samples [28,29,30,74]. However, to date, only bacteria and fungi that can completely mineralize or degrade methomyl have been isolated and characterized, while actinomycetes and algae that can degrade methomyl have not been isolated (Table 3).

It is commonly the case that a single strain can completely degrade methomyl [25]. *Stenotrophomonas maltophilia* M1, which was isolated from an irrigation site in Egypt, used 100 mg L^−1^ of methomyl as a carbon source and tolerated up to 1000 mg L^−1^ of methomyl in the presence of 0.05% glucose [30]. *Paracoccus* sp. mdw-1 was reported to completely degrade 100 mg L^−1^ of methomyl within 10 h at a pH of 7.0 and 30 °C [25]. *Pseudomonas* sp. EB20, which was isolated from water contaminated with persistent organic pollutants, degraded 77% of 10 mg L^−1^ of methomyl [43]. *Bacillus cereus*, *B. safensis*, *Pseudomonas aeruginosa* KT2440, *Novosphingobium* sp. FND3, and *Paracoccus* sp. YM3 efficiently removed 80% methomyl within 7 days as compared to the 40-day degradation period of *Flavobacterium* and *Alcaligenes* [76,77,85,87]. Interestingly, some bacteria can degrade methomyl as well as other pesticides, such as aldicarb, oxamyl, fenamiphos, and Imidacloprid [75,79,84,86]. Fungi have been proven to be potential degrading micro-organisms in nature [88,89]. Recently, fungi have received a considerable amount of attention due to their growth and extracellular enzymatic properties. Fungi not only have an extensive mycelium network and low specificity with respect to degrading enzymes, but also contain different enzymes, such as laccase, peroxidase, and dehydrogenase [90,91,92]. *Phanerochaete crysosporium*, which belongs to the white-rot fungal group, is one of the most effective fungal strains and can degrade a wide range of pesticides, aromatic hydrocarbons, and other xenobiotics [89,93]. Fungal bio-fortification is a method for improving the biosynthesis performance of pesticides, and *Trametes versicolor* was employed to efficiently degrade methomyl [74,94]. A versatile fungus, *Ascochyta* sp. CBS 237.37, was isolated to degrade methomyl, carbaryl, carbofuran, and carbofuran [88]. In addition, two strains of genetically engineered bacteria have also been successfully used to degrade methomyl [85,86]. Taking into account the contamination of the environment with various pesticides and the adaptability of indigenous micro-organisms to the environment, genetic engineering techniques may accelerate the application of degrading micro-organisms in situ.

Sometimes, single strains are not capable of complete degradation or have a weak degradation ability. In these cases, degradation can be mutually promoted by a co-culture or co-metabolism to enhance the enzyme activity. Bacteria that co-exist have a higher biodegradation ability than the individual species alone. Zhang et al. [29] isolated two bacterial strains, MDW-2 and MDW-3, from wastewater sludge samples and identified them as *Aminobacter* sp. and *Afipia* sp., respectively. Studies on their ability to degrade methomyl revealed that strain MDW-2 only accumulated intermediates and could not completely mineralize methomyl, whereas strain MDW-3 was unable to degrade methomyl. However, the combination of these two strains completely mineralized methomyl at a concentration of 50 mg L^−1^ within 3 days through co-metabolism. The five white-rot fungal strains WR1, WR2, WR4, WR9, and WR15 were isolated from horticultural soils through enrichment and screened for the ability to degrade methomyl. Degradation studies demonstrated that a single strain took 100 days to completely degrade 50 mg L^−1^ of methomyl whereas a combination of these strains completely degraded it in 50 days [26]. In addition to contaminated soil or water samples, pesticide-degrading bacteria can also be isolated from biological samples. Kawakami et al. [28] isolated *Bacillus cereus*, *Bacillus* sp., and *Pseudomonas aeruginosa* from human stool samples. These mixed bacterial strains possess an exceptional ability to degrade methomyl degradation and decompose it into dimethyl disulphide (DMDS) inside the human body. Roy and Das [84] achieved a microbial consortium of *Cupriavidus, Achromobacter*, and *Pseudomonas genera*, and showed that it can degrade high concentrations of carbamates, including methomyl, carbofuran, aldicarb, and methiocarb, in batch bioreactors. Methomyl can accumulate in rivers and, therefore, biofilms on the surface of rivers can produce methomyl-degrading microbes. Two microbial consortiums isolated from natural river biofilms were shown to remove methomyl or other carbamates and, thus, can be applied to purify rivers [79]. Mixed bacterial populations and microbial consortiums can also be applied in sewage treatment systems via activated sludge technology for the degradation of methomyl and its intermediates [75].

## 5. Molecular Mechanism of Methomyl Degradation

Methomyl degradation is linked to the genetic structure of micro-organisms. Each methomyl-degrading micro-organism has functional genes encoding for the enzymes that play a direct role in methomyl degradation. These enzymes can convert each metabolite into a nontoxic intermediate. Under adverse conditions, microbes benefit from methomyl as a source of nutrition. Previous studies have found that an enzymatic degradation system is more effective than the direct use of micro-organisms [95,96,97,98,99,100,101]. Genes and enzymes involved in the development of drugs have been investigated [102,103,104]. However, there are only a few studies on the enzymatic degradation pathway of methomyl.

Plasmids determine the degradation effect of bacteria and facilitate their study at the molecular level [105,106,107,108,109]. The PMb plasmid (5 KB) was isolated from *Stenotrophomonas maltophilia* M1 and screened for the ability to degrade methomyl via transformation into *Escherichia coli* [30]. Kulkarni and Kaliwal [86] isolated a plasmid from *E. coli* that can efficiently degrade methomyl. Furthermore, a carbamate–hydrolase gene *cehA* was isolated from *Pseudomonas* that controls the degradation of methomyl. Kulkarni and Kaliwal [80] also found that the plasmid of *Pseudomonas aeruginosa* controls the degradation of methomyl and can be used as a cloning vehicle in recombinant DNA technology. Another methomyl-degrading *E. coli* plasmid was isolated from the main chromosome [86]. Catalase and cytochrome oxidase were isolated from flavobacteria and alkaline bacteria, respectively; however, further studies on these degradation products were not carried out [83].

**Figure 4:**
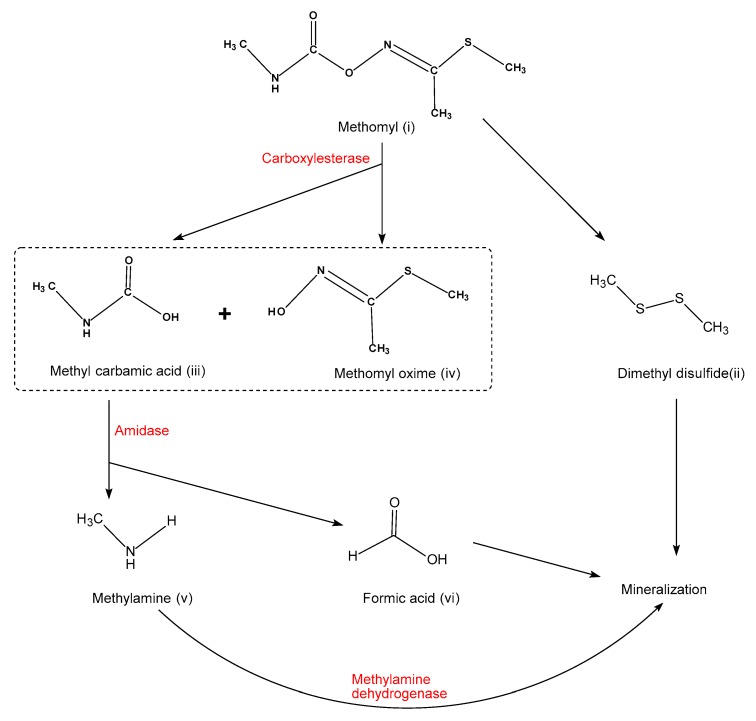
Proposed microbial degradation pathways of methomyl, adapted from [28,29,83].

Methomyl biodegradation pathways are presented in Figure 4. The methomyl degradation process includes hydroxylation, oxidation, and the cleavage of ester, C-N, C-S, and N-O bonds. Cleavage of an ester bond leads to the production of methyl carbamic acid (ⅲ) and methomyl oxime (ⅳ), which are catalyzed by carboxylesterase [83]. Then, methyl carbamic acid (ⅲ) will be broken down into formic acid (ⅴ) and methylamine (ⅵ), because amidases will attack the C-N bonds. Finally, formic acid (ⅴ) generates CO_2_, and methylamine (ⅵ) is degraded into formaldehyde and other minerals by methylamine dehydrogenase [83]. Fungal degradation of methomyl produces dimethyl disulfide (DMDS) (ⅱ) through the cleavage of C-S bonds [28]. Degradative plasmids also play an important role in degradation studies of various pesticides. Unlike the physicochemical degradation pathways, dimethyl disulfide (ⅱ) is formed during fungal biodegradation.

However, more focused research is needed to culture and identify micro-organisms with potent catabolic genes and enzymes and explore novel metabolic pathways that can act on a variety of pesticides.

## 6. Conclusions and Future Perspectives

Methomyl plays a very important role in modern agricultural practices, but its toxicity has raised widespread concern. Recently, different physicochemical methods have been developed for the removal of methomyl from contaminated environments, but they are expensive to use and generate toxic intermediate products. Thus, microbial degradation of methomyl is considered to be the most effective method. A few methomyl-degrading bacteria have been isolated, including *Paracoccus*, *Pseudomonas*, and *Aminobacter*. However, methomyl degradation pathways and related degradative enzymes and functional genes have not been thoroughly explored. Therefore, advanced molecular techniques, such as metagenomics, proteomics, and transcriptomics, should be developed to perform a genetic analysis of methomyl-degrading enzymes and catabolic genes, missing links, and degradation evolution mechanisms and pathways. A better understanding of the detoxification pathways in non-target species may help us to design safer and more specific carbamate insecticides. Natural micro-organisms lack the ability to simultaneously degrade different types of pesticides; however, synthetic biology offers powerful tools to create multifunctional biodegrading micro-organisms for in situ bioremediation. In the future, genetically engineered micro-organisms for methomyl degradation and related genes and enzymes should be explored in depth. DNA stable isotope probing techniques can be used to assess which organisms are degrading methomyl in situ, as the indigenous organisms may be better adapted than isolates.

## Figures and Tables

**Table 1:** Toxicological studies of methomyl in humans and animals.

S.No.	Study Sample/Sample Sources	Concentration/Volume of Methomyl	Specific Statement	References
1	Tilapia	3.2-10 mg L^−1^	Genotoxicity caused by methomyl	[35]
2	Tilapia	0.2-200 µg L^−1^	Injury to and apoptosis of testicular tissue	[36]
3	Tilapia	0.2-200 μg L^−1^	Inhibition of the antioxidant system	[37]
4	Tilapia	0.2-200 μg L^−1^	Disruption of the endocrine system and genetic variation	[38]
5	Frogs	8.69 mg L^−1^	Reduced growth rates and tissue damage	[16]
6	Frogs	10 mg L^−1^	Methomyl induces teratogenicity and neurotoxicity	[17]
7	Frogs	15.43 mg L^−1^	Death of or deformations in tadpoles	[39]
8	Rats	17 mg kg^−1^	Inhibition of the reproductive system	[40]
9	Rats	0.25-2.5 mg kg^−1^	Inhibited activity of brain ChE and RBC ChE	[41]
10	Rats	0.5-20 mg kg^−1^	Inhibition of the reproductive system	[42]
11	Rats	10 mg kg^−1^	Inhibition of liver function and enzyme activity	[43]
12	Human	unknown	17 people poisoned (2012–2016, France)	[18]
13	Human	570 μg L^−1^	Death by inhalation of too much methomyl	[19]
14	Human	Unknown	The person died after swallowing methomyl	[20]
15	Human	300 cm^3^	Reversible cortical blindness and continuous peeling	[21]
16	Cells	6-30 mmol L^−1^	DNA damage and apoptosis induced by methomyl	[44]
17	Zooplankton and fish	8 μg L^−1^	Reduction in the efficiency of the food chain in a Cr/Dg system	[34]

**Table 2:** Physical and chemical approaches to the removal of methomyl from contaminated environments.

S.No.	Study Sample/Sample Sources	Physicochemical Method Used	Specific Statement	References
1	UV/TiO_2_	Photocatalysis	100% methomyl was degraded in 45 min	[60]
2	Photo-Fenton	AOP_S_	100% methomyl was degraded in an hour	[22]
3	Fenton/Fe-ZSM-5	Photocatalysis	Methomyl was completely degraded	[57]
4	Fenton/H_2_O_2_/UV	AOP_S_	Methomyl was degraded within 320 min	[43]
5	Fenton/HA	Photocatalysis	HA promotes the degradation of methomyl	[64]
6	Activated Carbon	Adsorption	Methomyl was removed in 2.5 h	[58]
7	US/Photo-Fenton	AOP_S_	Promotion of the degradation ability	[24]
8	TiO_2_ nanoparticles	Photocatalysis	Pesticide was removed in 1 h	[63]
9	UV/TiO_2_	AOP_S_	Promotion of the degradation ability	[65]
10	HC/H_2_O_2_	AOP_S_	Promotion of hydrodynamic cavitation	[23]
11	O_3_/UV	AOP_S_	UV can promote the degradation effect	[61]
12	HC/Fenton/O_3_	AOP_S_	Promotion of methomyl degradation	[66]
13	DSA Ti/RuO_2_ electrode	Electrocatalysis	90% methomyl was degraded within 0.5 h	[59]

**Table 3:** Microbial degradation of methomyl.

S.No.	Strain Or Community	Sample Source	Detected Metabolites	Comments	References
1	Mixed microbial community	Activated sludge from a domestic wastewater treatment plant	Methomyl oxime	Methomyl and its intermediates were completely degraded on the 12th and the 28th day, respectively	[75]
2	*Novosphingobium* SP. FND3	No data	No data	Degraded 63% methomyl within 16 h	[76]
3	*Paracoccus* sp. YM3	Sludge from a wastewater treatment facility	No data	Strain removed more than 80% of methomyl (50 mg L^−1^) in 7 days	[77]
4	*Stenotrophomonas maltophilia* M1	Irrigation sites in Egypt	No data	Bacteria can grow on methomyl (100 mg L^−1^) and can tolerate up to 1000 mg L^−1^ of methomyl in the presence of 0.05% glucose	[30]
5	*Paracoccus* sp. mdw-1	Methomyl wastewater treatment plant	Methomyl oxime	100 mg L^−1^ of methomyl was transformed into an unknown metabolite within 10 h	[25]
6	White-rot fungal isolates WR1, WR2, WR4, WR9, and WR15	Rift-valley region and a Mountain region in Kenya	No data	Complete degradation of 50 mg L^−1^ of methomyl by a single strain in 100 days whereas mixed strains took only 50–60 days	[26]
7	*Pseudomonas* sp. EB20	Water polluted by persistent organic pollutants in Egypt	No data	77% of 10 mg L^−1^ of methomyl was degraded within 2 weeks	[43]
8	*Flavobacterium*, *Alcaligenes*	Horticultural farms in Rift Valley and Central Kenya	No data	Strains completely degraded methomyl and its metabolites within 40 days as compared to the control	[78]
9	A consortium of *Gomphonema parvulum*, *Cymbella silesiaca*, and *Nitzschia dissipata*	Tseng-Wen River	No data	Methomyl was efficiently removed by biofilms containing degrading micro-organisms and diatoms	[79]
10	Microbial communities	Natural river biofilms	No data	91% of added methomyl (50 mg L^−1^) was removed in 7 days	[27]
11	*Pseudomonas aeruginosa*	Soil samples from Dharwad	No data	Methomyl was significantly decreased	[80]
12	*Serratia plymuthica*	Marine coastal sediment	No data	Bacterium showed an excellent ability to remove imidacloprid, methomyl, and fenamiphos	[81]
13	*Bacillus cereus*, *Bacillus safensis*	Pesticide-treated crop field in India	No data	*B. cereus* and *B. safensis* showed 88.25% and 77.5% of methomyl degradation, respectively, within 96 h	[82]
14	*Pseudomonas*	Banana plantation, Greece	No data	Transformed all tested carbamates including aldicarb and methomyl	[83]
15	*Bacillus cereus*, *Pseudomonas aeruginosa*	Human stool samples provided by volunteers	Dimethyl disulfide	Strains can generate large quantities of DMDS	[28]
16	*Trametes versicolor*	No data	No data	More than 99% methomyl was removed by the bioaugmentation of the strain	[74]
17	A consortium of *Cupriavidus*, *Achromobacter* and *Pseudomonas genera*	Biopurificati-on system	No data	Methomyl was completely degraded within 7 days	[84]
18	*Aminobacter* sp. MDW-2 and *Afipia* sp. MDW-3	Wastewater treatment system of a pesticide manufacturer	Methomyl oxime, methyl carbamic acid	Strains MDW-2 and MDW-3 co-existed and completely degraded 50 mg L^−1^ of methomyl within 3 days	[29]
19	*Pseudomonas putida* KT2440	Genome editing	No data	Strain simultaneously degraded organophosphates, pyrethroids, and carbamates	[85]
20	*Escherichia coli*	India	No data	Methomyl was efficiently degraded by *Escherichia coli* with a plasmid	[86]
21	*Bacillus cereus*, *Bacillus safensis*	No data	No data	Strains degraded methomyl, carbendazim, and imidacloprid in NB medium	[87]
22	*Ascochyta* sp. CBS 237.37	Paddy and maize cultivated fields, India	No data	Strain removed 90.15% of 85 mg L^−1^ of carbamates in 40 days	[88]
